# Interaction between Allee effects caused by organism-environment feedback and by other ecological mechanisms

**DOI:** 10.1371/journal.pone.0174141

**Published:** 2017-03-23

**Authors:** Lijuan Qin, Feng Zhang, Wanxiong Wang, Weixin Song

**Affiliations:** 1 College of Resources and Environmental Science, Gansu Agricultural University, Lanzhou, China; 2 Center for Quantitative Biology, College of Science, Gansu Agricultural University, Lanzhou, China; Shanxi University, CHINA

## Abstract

Understanding Allee effect has crucial importance for ecological conservation and management because it is strongly related to population extinction. Due to various ecological mechanisms accounting for Allee effect, it is necessary to study the influence of multiple Allee effects on the dynamics and persistence of population. We here focus on organism-environment feedback which can incur strong, weak, and fatal Allee effect (AE-by-OEF), and further examine their interaction with the Allee effects caused by other ecological mechanisms (AE-by-OM). The results show that multiple Allee effects largely increase the extinction risk of population either due to the enlargement of Allee threshold or the change of inherent characteristic of Allee effect, and such an increase will be enhanced dramatically with increasing the strength of individual Allee effects. Our simulations explicitly considering spatial structure also demonstrate that local interaction among habitat patches can greatly mitigate such superimposed Allee effects as well as individual Allee effect. This implies that spatially structurized habitat could play an important role in ecological conservation and management.

## Introduction

Allee effect, the positive relationship between per capita growth rate (individual’s fitness) and population density at low population density [1], has received considerable attention in ecology and conservation because it is directly related to population extinction [2–4] and can incur complicated spatial pattern [5, 6]. Many mechanisms may account for Allee effect such as organism-environment feedback [7, 8], mate-finding limitations [9–11], cooperative defense [12, 13], social dysfunction [1, 14, 15], inbreeding depression [16, 17], and predator avoidance or defense [1, 18, 19]. Allee effect also occurs at a metapopualtion level similar to local populations, which may be because of dispersal cost or colonization difficulty [15, 20–23]. Due to various ecological mechanisms leading to Allee effect, it is necessary to study the influence of multiple Allee effects on population dynamics and persistence. Berec et al. [24] has suggested that two or more Allee effects could affect the dynamics of a population, and more empirical evidence for multiple Allee effects have been provided [10]. We here studied organism-environment positive feedback when Allee effect caused by other ecological mechanism is involved in also.

Organism-environment positive feedback (i.e. habitat modification caused by organism) is an important ecological mechanisms leading to Allee effect [7, 25]. Growth, survival and metabolism of organisms are limited by the physical environments (e.g. temperature, wind, and turbulence) and chemical environments (e.g. oxygen, toxins, and hormones) [26]. Individuals in larger groups will live better if organisms can improve their environment in some ways [10, 27–29]. Such an organism-environment positive feedback is a crucial ecological process in ecosystems [27, 30], which could lead to Allee effect because the fitness of a population may indirectly depend on population density through environmental intermediary [7, 8, 31, 32]. However, the dynamics and consequence of the organism-environment feedback is still unclear when the population is also subjected to Allee effect by other ecological mechanism.

In this paper, we therefore investigate multiple Allee effects respectively caused by the organism-environment feedback (AE-by-OEF) and other ecological mechanisms (AE-by-OM), and specifically analyzed how the superimposed and interacted Allee effects affect the dynamics and persistence of population. Through simulations considering explicitly spatial structure, we also demonstrate the effects of spatial structure on Allee effects.

## Models

### Mean-field model

With mean-field assumption, the organism-environment positive feedback in patchy habitat can be described by following ordinary equations [8]

$$\begin{array}{lll} \frac{\text{d}p}{\text{d}t} & = & cp(h-p)-(e+d)p\\ \frac{\text{d}h}{\text{d}t} & = & (\lambda p+\mu )(1-h)-dh \end{array}.$$ (1)

The variable *p* is the fraction of patches occupied by the species, and *h* is the fraction of suitable patches (thus 1 − *h* indicates the fraction of unsuitable patches, or the fraction of habitat loss), where obviously *p* ≤ *h*. Parameter *c* and *e* are the colonization and extinction rate, respectively; *d* is the habitat destruction rate due to human activities or natural causes; *λ* and *μ* are the internal (organismic) and external habitat restoration rate, respectively. Parameter *c*, *e*, *d*, *λ* and *μ* are all non-negative constants. The system (Eq 1) returns to the classical Levins model when *λ* = 0 [33]. The organism-environment positive feedback, reflected by parameter *λ* > 0 in Eq 1, can generate Allee effect (i.e. AE-by-OEF, [8, 27, 34]).

To investigate how the AE-by-OEF functions when the population is also subjected to Allee effect by other mechanism (AE-by-OM), we follow classical method to incorporate AE-by-OM into Eq 1 [11, 35–37].

$$\begin{array}{lll} \frac{\text{d}p}{\text{d}t} & = & cp(h-p)\frac{p}{p+a}-(e+d)p\\ \frac{\text{d}h}{\text{d}t} & = & (\lambda p+\mu )(1-h)-dh \end{array}.$$ (2)

The term *p* / (*p* + *a*) indicate AE-by-OM, where *a* ≥ 0 denotes the intensity of the Allee effect [23, 35–38]. In particular *a* = 0 excludes AE-by-OM (Eq 2 returns to Eq 1), and *λ* = 0 means AE-by-OEF disappears (see Results). Our model (Eq 2) thus potentially includes the two types of Allee effects when *a* > 0 and *λ* > 0.

### Probability transition model

In order to reveal the effect of spatial structure on Allee effect, we constructed probability transition model (PTM) based on Markov process, which captures the dynamical change of state probability of each patch over time depending on the state of local neighboring patches. Because there are all three possible states for each patch in our modeling system (i.e. occupied, suitable but empty, and unsuitable), we can only focus on two independent probabilities that a patch is occupied or suitable. Specifically, the dynamical changes of the two probabilities for patch *i* were given by [39]

$$\begin{array}{lll} \frac{dp_{i}}{dt} & = & c_{i}{\tilde{p}}_{i}(h_{i}-p_{i})\frac{{\tilde{p}}_{i}}{{\tilde{p}}_{i}+a}-(e+d)p_{i}\\ \frac{dh_{i}}{dt} & = & (\lambda {\tilde{p}}_{i}+\mu )(1-h_{i})-dh_{i} \end{array}$$ (3)

where *p*_*i*_ represents the probability that patch *i* is occupied, and *h*_*i*_ the probability that patch *i* suitable. The notation ${\tilde{p}}_{i}$ is the average probability that neighboring patch of patch *i* is occupied, i.e. ${\tilde{p}}_{i}=\frac{1}{z}\sum p_{j}$, where *p*_*j*_ is the probability that a neighboring patch *j* of patch *i* is occupied and *z* neighborhood size (*z* = 4 called von Neumann neighborhood and *z* = 8 Moor neighborhood). In particular Eq 3 will return to Eq 2 when we average *p*_*i*_ and *h*_*i*_ over all patches with infinite neighborhood size. This shows that Eq 3 can not only handle situations with various neighborhood sizes but also includes Eq 2. Because it is very difficult to solve the set of ordinary equations (Eq 3) because of its extremely high dimension, we numerically analyzed it on lattice space with periodic boundary.

### Cellular automata model

Using a cellular automata model (CAM) with considering stochasticity [12, 40], we also simulated the system mentioned above on lattice space. Each patch is either occupied (denoted by 2), empty but suitable (denoted by 1), or unsuitable (denoted by 0). We define state transition rule (i.e. update rule) corresponding to above models. An unsuitable patch becomes suitable (0 → 1) at rate $\lambda \tilde{p}+\mu$ (habitat restoration), where $\tilde{p}$ represents the fraction of occupied patches in its neighborhood; an empty but suitable patch becomes unsuitable (1 → 0) at rate *d* (habitat destruction) or occupied (1 → 2) at rate $c\tilde{p}\tilde{p}/(\tilde{p}+a)$ (recolonization); and an occupied patches becomes unsuitable (2 → 0) at rate *d* (habitat destruction) or empty but still suitable (2 → 1) at rate *e* (local extinction). The update rules are summarized in Table 1. We used the periodic boundary condition and synchronous update in our simulations [12, 41].

**Table 1:** Transition rate for the lattice model.

State transition	Rate
0 → 1	$\lambda \tilde{p}+\mu$
1 → 0	*d*
1 → 2	$c\tilde{p}\frac{\tilde{p}}{\tilde{p}+a}$
2 → 0	*d*
2 → 1	*e*

**Note:** Here 0, 1, and 2 denotes that a patch is unsuitable, suitable but empty, or occupied, respectively. $\tilde{p}$ represents the fraction of occupied patches in neighborhood. Other parameters are the same as in the text.

## Results

**Fig 1:**
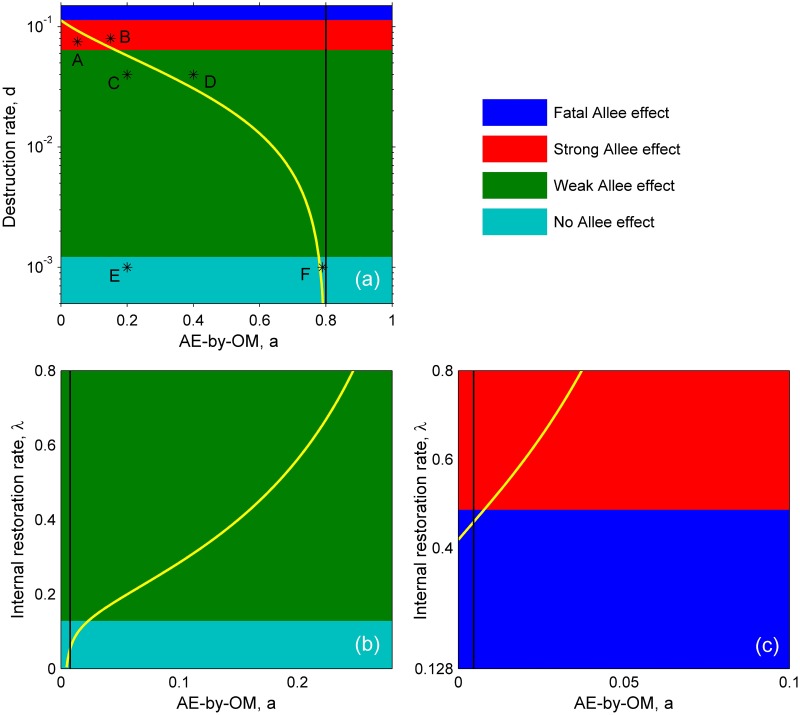
Interaction between organism-environment positive feedback and Allee effect caused by other ecological mechanisms. Organism-environment feedback results in weak, strong, and fatal Allee effect as well as the situation of no Allee effect (denoted by green, red, blue, and cyan area, respectively). Allee effect caused by other ecological mechanisms is strong (left of black line) and fatal (right of black line) Allee effect. The interaction of the two kinds of Allee effects leads to strong (below yellow line in (a), above yellow line in (b) and (c)) and fatal (above yellow line in (a), below yellow line in (b) and (c)) Allee effect. For the parameters, We take: *μ* = 0.03 and *e* = 0.1 in (a)-(c); *c* = 0.5 in (a) and (b); *d* = 0.05 in (b) and (c); *λ* = 0.8 in (a); *c* = 0.3 in (c); Point A-F in (a) correspond to panel A-F in Fig 3.

**Fig 2:**
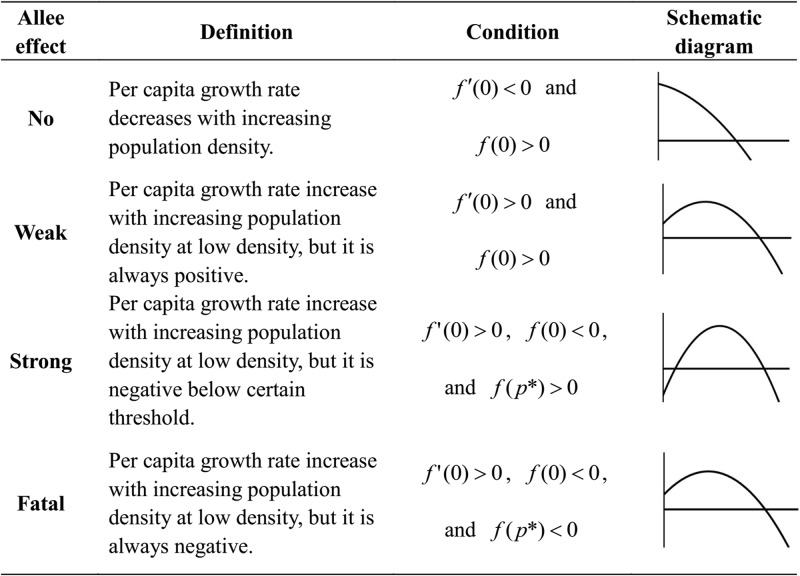
Definitions of various Allee effects. **Note:** function *f*(*p*) is per capita growth rate of population, and p* > 0 its maximum point. In schematic diagram, the horizontal axis is population size or density, and vertical axis is per capita growth rate of population.

**Fig 3:**
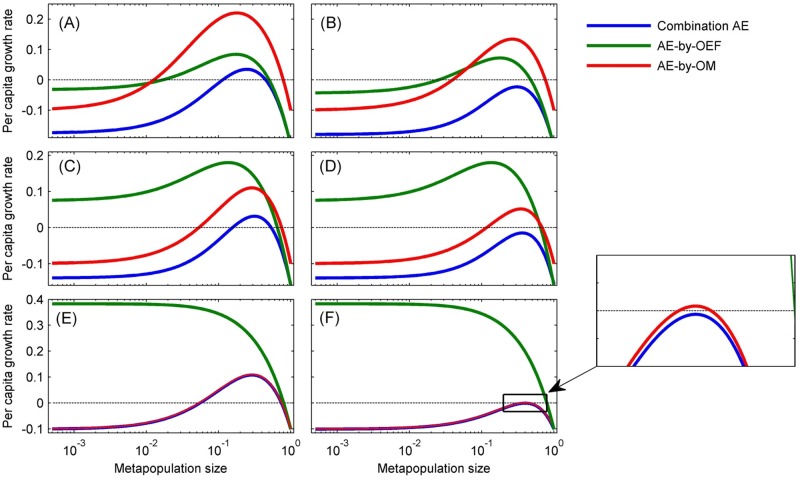
Relationship between population size and per capita growth rate. Green and red lines represent organism-environment feedback (*a* = 0) and Allee effect caused by other mechanism (*d* = 0), respectively; blue lines represent the combination of the two types of Allee effects. Panel A-F corresponds to point A-F on Fig 1a.

**Fig 4:**
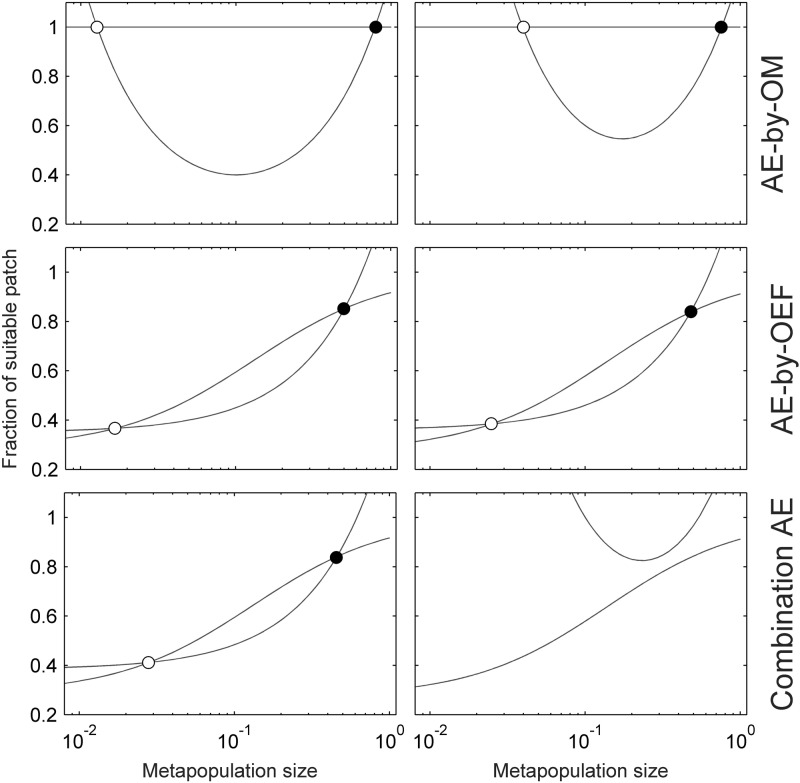
Phase plane analysis. The curves indicate zero isoclines of corresponding system. Filled and empty circles represent stable and unstable equilibriums, respectively. The left column correspond to Fig 3A and 3B (also point A and B in Fig 1a).

Organism-environment positive feedback will individually incur Allee effect (i.e. AE-by-OEF) when *λ* > (*μ* + *d*)^2^ / *d*, of which inherent characteristic depends on model parameters (Eqs 1 or 2 with *a* = 0). For example, the population will be subjected to weak, strong, and fatal Allee effect in turn with increasing habitat destruction rate *d* (see Table 2, Figs 1 and 2). Individual AE-by-OM in our model is strong when *a* < (*δ*–*β*/(*β* + 1))^2^/(4*δ*) and *δ* < *β*/(*β* + 1) (where *δ* = (*e* + *d*)/*c*, *α* = *λ*/*d*, *β* = *μ*/*d*), and fatal otherwise (Fig 1). This system becomes more complicated when the two types of Allee effects are superimposed and interacted. Through analyzing per capita growth rate when habitat dynamics is at equilibrium (i.e. d*h*/*dt* = 0), we found that the combinations of strong AE-by-OM and either strong or weak AE-by-OEF, or even organism-environment feedback without Allee effect (*λ* > (*μ* + *d*)^2^/*d*), are all able to lead to strong Allee effect with higher Allee threshold (Fig 3A, 3C and 3E). These combinations can also incur fatal Allee effect which means population extinction in any case (Fig 3B, 3D and 3F). Phase plane analysis also confirmed these results (Fig 4).

**Table 2:** Condition for Allee effect caused by organism-environment positive feedback.

Characteristic of Allee effect	Parameter condition
**No**	$\lambda <\frac{{\left(\mu +d\right)}^{2}}{d},\delta <\frac{\beta }{\beta +1}$
**Weak**	$\lambda >\frac{{\left(\mu +d\right)}^{2}}{d},\delta <\frac{\beta }{\beta +1}$
**Strong**	$\lambda >\frac{{\left(\mu +d\right)}^{2}}{d},\frac{\beta }{\beta +1}<\delta <\frac{{\left(\sqrt{\alpha }-1\right)}^{2}+\beta }{\alpha }$
**Fatal**	$\lambda >\frac{{\left(\mu +d\right)}^{2}}{d},\delta >\frac{{\left(\sqrt{\alpha }-1\right)}^{2}+\beta }{\alpha })$

Note: *δ* = (*e* + *d*)/*c*, *α* = *λ*/*d*, *β* = *μ*/*d*)

The stability analysis also roots for the above results (Appendix). There is only one boundary equilibrium (0, *β* / (*β* + 1)) for both Eqs 1 and 2, which represents population extinction. The equilibrium is stable for Eq 1 if *δ* > *β* / (*β* + 1) and unstable otherwise, while it is always stable for Eq 2 (Appendix). For the both equations, when the boundary equilibrium is stable, the two equations have either two interior equilibriums (the smaller unstable and the larger stable) or no interior equilibrium (Appendix), which means strong or fatal Allee effect. When the boundary equilibrium is unstable for Eq 1, the equation has unique interior equilibrium, meaning weak Allee effect or no Allee effect (Table 2). Totally, multiple Allee effects either enlarge Allee threshold or change the inherent characteristic of Allee effect, and such enlargement and change will be enhanced dramatically with increasing individual Allee effects.

**Fig 5:**
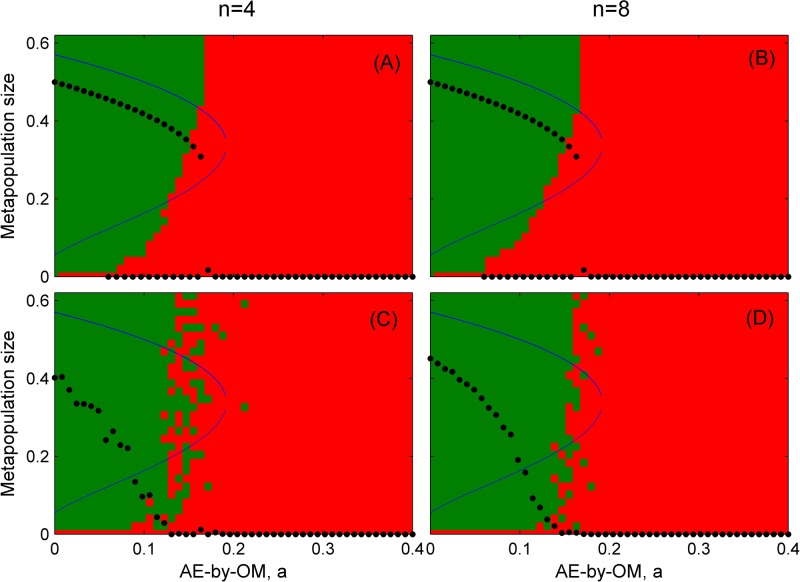
The dependence of population dynamics on its initial state when population dispersal locally. (A) and (B) based on probability transition model, (C) and (D) based on cellular automata model (neighborhood size *n* = 4 for A and C, *n* = 8 for B and D). Red areas indicate the initial values leading to the population extinction, while green areas represent the initial values from which the population persists stably. The simulations ran on 100 × 100 lattices with periodic boundary condition. Parameter *λ* = 0.4, *μ* = 0.1, *d* = 0.1; and other parameters are the same as in Fig 1a.

**Fig 6:**
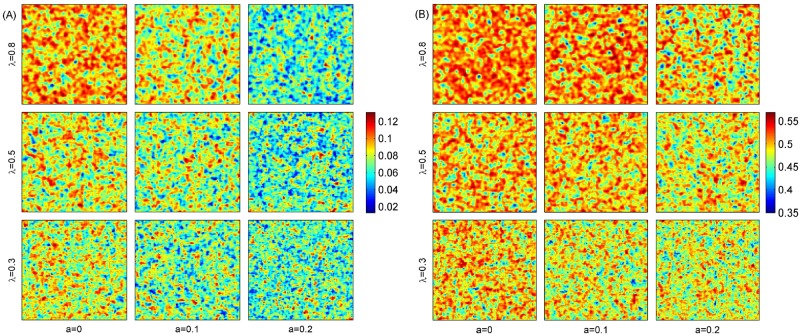
The distribution of population subjected to Allee effects on two-dimensional space, which are snapshots of 100 × 100 lattice when simulations arrive at 3000 time step. The color hot indicates the probability of patch occupancy (A) and suitable patch (B). Parameters: *d* = 0.1 and other parameters are the same as in Fig 1a.

**Fig 7:**
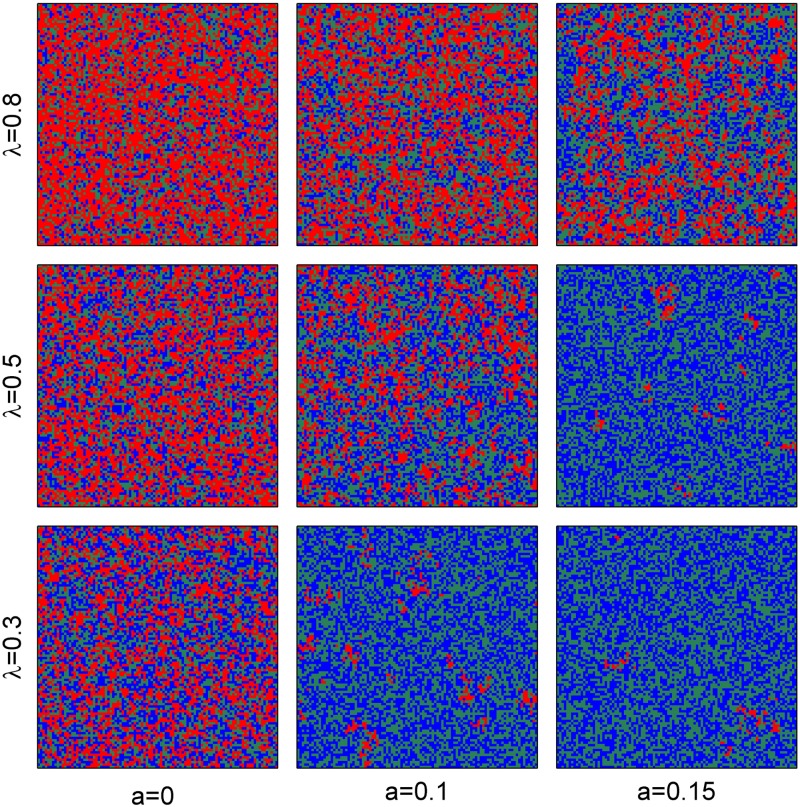
Spatial distribution of population subjected to Allee effect. Red, green, and blue colors indicate occupied cells, suitable but no occupied cells, and destroyed (unsuitable) cells. The simulations ran on 100 × 100 lattices with periodic boundary condition and 4 neighborhood. Parameters are taken as *a* = 0, 0.1, and 0.15 from left to right, *λ* = 0.3, 0.5, and 0.8 from bottom to top, respectively. Other parameters values are the same as in Fig 4.

Our simulations based on both PTM and CAM with explicitly spatial structure demonstrated that spatially local interaction between organism and environment can make both individual and superimposed Allee effects mitigate greatly and even disappear (see Fig 5, notably *a* = 0 and *λ* = 0 respectively represent individual AE-by-OEF and AE-by-OM). The spatial heterogeneity of population distribution could play important role for the consequence of the local effect. Although low-density local regions are subjected to Allee effect and make sub-populations extinct, high-density local regions save the population globally. In our simulations we obviously observed the aggregated distribution pattern with distinct spatial boundary not only for population but also suitable habitat (Figs 6 and 7). However, because colonization and habitat restoration incurred by organism are limited, local interaction leads to lower global population density than the predictions from mean-field model (Fig 5). This shows that spatially limited interaction is good for preventing population extinction although it decreases population density potentially.

## Discussion

Organism-environment positive feedback is ubiquitous in real world, especially in severe environment such as dry land [42–44] and intertidal zone [27]. Allee effect is a distinct feature caused by the positive feedback. Due to diverse mechanism leading to Allee effect, it is necessary to estimate the effect of Allee effect caused by the organism-environment feedback on population dynamics and persistence when another mechanism generating Allee effect also act on the population [10, 24]. We here study theoretically the interaction of the two type of Allee effect to reveal their effect on population dynamics and persistence. The combination of multiple Allee effects could largely increase the extinction risk of population either due to the enlargement of Allee threshold or the change of inherent characteristic of Allee effect (Fig 3). We further found that the spatial range of the organism-environment feedback also profoundly affect the appearance of Allee effect. Both combined and individual Allee effect greatly mitigates when interaction is limited locally (Fig 5). This implies spatial scale could play an important role in ecological conservation [12, 15, 39, 45].

Mathematical models are useful in helping to understand the dynamic processes of involved populations and in making practical predictions [46–49]. In general, we can classify modeling methodologies for Allee effect into two distinctive categories: phenomenological model and mechanistic model. The former directly incorporates Allee effect into the dynamic models of populations, and the modelers are often interested in the consequence of Allee effect (e.g. [35, 36, 50]); the later models the underlying mechanism that incurs Allee effect, and generally tests how Allee effect is generated (e.g. [8, 51, 52]). In our model, organism-environment feedback which can result in Allee effect was described in details (i.e. mechanistic modeling), while another Allee effect was phenomenologically modeled using a frequently-used classical method (Eqs 1 & 2). Therefore, our models totally include the two kinds of modeling methodologies for the two types of Allee effects.

Much attention has been paid to Allee effect not only because it is an important ecological problem itself, but also it is directly related to population extinction and thus significant to ecological conservation [53, 54]. Recent years, multiple Allee effects, resulting from various mechanisms but acting on the same population, have become a hot point for study, with examples from plants, invertebrates and vertebrates, from natural and exploited populations, and from terrestrial and marine ecosystems [24, 55]. Mathematical models can also increase our understanding of the interaction among multiple Allee effects [10, 24]. We here constructed a set of mathematical models for organism-environment feedback system subjected to Allee effect from other mechanism, without and with spatial structure. Agreeing with the results of Berec et al. [24], our model demonstrated that multiple Allee effects could be stronger than the simple sum of involved individual Allee effects (Fig 3). This implies that the interaction of multiple Allee effects could be nonlinear (i.e. nonadditivity), thus we need to carefully refer to those predictions from linear models in conservation and management.

Reaction-diffusion equations are used traditionally to model the spatial dynamics of populations [5, 6, 56–62]. However, it is difficult to handle various neighborhood sizes (e.g. changeable dispersal kernel) for them. We thus constructed our model system based on lattice space in order to conveniently assess the ecological significance of spatial scale [39]. Spatial scale is an important ecological factor in ecology, which plays a key role in the coexistence of competitive species [39, 63–65], biodiversity persistence [66, 67], epidemic spread [68–70], evolutionary consequence [12, 71], pattern formation [13, 39, 72] and so on. We here found that spatial scale has important significance for the appearance of Allee effect. When organism-environment interaction is limited locally, Allee effect will mitigate although the global size of population decrease (Fig 5). The emergence of spatial pattern resulting from local interaction could account for this mitigation of Allee effect (Figs 6 and 7). Although low-density local regions are subjected to Allee effect and make sub-populations extinct, high-density local regions save the population globally. Pattern formation could prevent population extinction [13, 39, 73].

## Appendix: Stability analysis for our system

When *a* = 0, the Eq 2 returns to Eq 1. When habitat dynamics is at equilibrium (i.e. d*h*/*dt* = 0), the per capita growth rate of population (i.e.$\frac{1}{p}\frac{\text{d}p}{\text{d}t}$) is

$$f(p)=c(\frac{\lambda p+\mu }{\lambda p+\mu +d}-p)-(e+d)$$ (A1)

From the condition of Fig 2, we deduce that organism-environment positive feedback will individually incur Allee effect (AE-by-OEF) when *λ* > (*μ* + *d*)^2^/*d*. We summarize parameter conditions of various Allee effects (Details are shown in Table 2). When *λ* = 0, the AE-by-OEF disappears and the population is only subjected to AE-by-OM in Eq 2. Through simple calculation, the interior equilibrium must be the solution of following equation

$$c(\frac{\mu }{\mu +d}-p)\frac{p}{p+a}-(e+d)=0.$$ (A2)

Thus, When *λ* = 0, Eq 2 has at most two interior equilibriums, and necessary conditions for the existence of two interior equilibriums are *a* < (*δ*–*β*/(*β* + 1))^2^/(4*δ*) and *δ* < *β*/(*β* + 1). When *δ* > *β*/(*β* + 1), there is no interior equilibrium.

When *λ* > 0 and *a* > 0, Eq 2 includes the two types of Allee effects. Eq 2 has one boundary equilibrium *E*_0_(0, *μ*/(*μ* + *d*)), and the eigenvalues of the Jacobian matrix at *E*_0_ are − (*e* + *d*) and − (*μ* + *d*). Thus, the boundary equilibrium *E*_0_(0, *μ*/(*μ* + *d*)) is locally asymptotically stable.

The zero isocline for d*p*/*dt* = 0, denoted by

$$C_{1}:\;h_{1}(p)=\delta (p+a)/p+p,$$ (A3)

where *δ* = (*e* + *d*)/*c*.

And the zero isocline for d*h*/*dt* = 0, denoted by

$$C_{2}:\;h_{2}(p)=\left(\lambda \text{p}+\mu \right)/\left(\lambda p+\mu +d\right).$$ (A4)

The interior equilibriums of system (2) are the points of intersections of two curves *C*_1_ and *C*_2_. It is convenient to express this as

$$p^{3}+Ap^{2}+Bp+C=0,$$ (A5)

where *A* = *δ*– 1 + (*μ* +*d*)/*λ*, *B* = *δ*(*μ* +*d*)/*λ +a*–*μ*/*λ*, *C* = *a δ*(*μ* +*d*)/*λ*.

In order to determine whether there exists interior equilibriums, let

$$f(p)=p^{3}+Ap^{2}+Bp+C.$$ (A6)

After direct computation, we have *f*(1) > 0, *f*(0) > 0, *f*′(1) > 0, *f*″(1) > 0, *f*‴(*p*) >0.

We can deduce that *f*″(*p*), *f*′(*p*) and *f*(*p*) are monotonically increasing functions for *p* ∈ (1, +∞). From the monotonicity of *f*(*p*), we can see that the Eq (A5) has no positive root when *p* > 1. That is to say, all positive solutions of Eq (A5) exist in (0, 1).

Note Δ = *A*^2^ – 3*B*, if Δ ≤ 0, *f*(*p*) is strictly increasing in (0, +∞) since *f*′(*p*) ≥ 0, which yields *f*(*p*) ≥ *f*(0) = *C* > 0. And Eq (A5) has no positive root.

If Δ > 0, then the equation *f*′(*p*) = 0 has two roots, $p_{1}=\left(-A-\sqrt{\Delta }\right)/3,$ $p_{2}=\left(-A+\sqrt{\Delta }\right)/3\;(p_{1}<p_{2})$. It is obvious that *f*(*p*) is strictly decreasing in (*p*_1_, *p*_2_) and increasing in (−∞, *p*_1_) and (*p*_2_, +∞).

In addition, we get

$$f(p_{1})=\frac{1}{3}(3f(p_{1})-p_{1}f^{\prime }(p_{1}))=\frac{A(2A^{2}-9B)+2\sqrt{\Delta^{3}}}{27}+\frac{a(\mu +d)}{\lambda };$$ (A7)

$$f(p_{2})=\frac{1}{3}(3f(p_{2})-p_{2}f^{\prime }(p_{2}))=\frac{A(2A^{2}-9B)-2\sqrt{\Delta^{3}}}{27}+\frac{a(\mu +d)}{\lambda }.$$ (A8)

Then, we can easily obtain the following results:

1. If *A* > 0, *B* > 0, and *A*^2^ > 3*B*, or *A*^2^ ≤ 3*B*, then system (2) has no positive equilibrium.
2. If either of the following inequalities holds: *B* < 0, *f*(*p*_2_) < 0 or *A* < 0, *B* > 0, *A*^2^ > 3*B*, *f*(*p*_2_) < 0, system (2) has two positive equilibriums.
3. If either of the following inequalities holds: *A* > 0, *B* < 0, *f*(*p*_2_) = 0; or *A* ≤ 0, *A*^2^ > 3*B*, *f*(*p*_2_) = 0, system (2) has an unique positive equilibrium.

Denote the right-hand sides of the two equations of system (2) by *f*(*p*, *h*) and *g*(*p*, *h*) respectively. At any interior (positive) equilibrium (p*, h*), we can write the Jacobian matrix as

$$J(p^{*},h^{*})={\left[\begin{array}{cc} f_{p} & f_{h}\\ g_{p} & g_{h} \end{array}\right]}_{\left(p^{*},h^{*}\right)}=\left[\begin{array}{cc} \left(ac\delta -c{\left(p^{*}\right)}^{2}\right)/\left(p^{*}+a\right) & c{\left(p^{*}\right)}^{2}/\left(p^{*}+a\right)\\ \lambda (1-h^{*}) & -(\lambda p^{*}+\mu +d) \end{array}\right],$$ (A9)

where subscripts denote partial derivatives. Obviously, three elements of *J* satisfy *f*_*h*_ > 0, *g*_*p*_ > 0 and *g*_*h*_ < 0.

Implicit differentiation of the equation for the *p*− null line, *f*(*p*, *h*_1_(*p*)) = 0, gives the slope

$${h^{\prime }}_{1}(p)=-f_{p}/f_{h}.$$ (A10)

Similarly, from *g*(*p*, *h*_2_(*p*)) = 0, we obtain

$${h^{\prime }}_{2}(p)=-g_{p}/g_{h}.$$ (A11)

We use Eqs (A10) and (A11) to write the determinant as $\text{det}(J)=f_{p}g_{h}-g_{p}f_{h}=f_{h}g_{h}({h^{\prime }}_{2}-{h^{\prime }}_{1})$. Then if *f*_*p*_ < 0 and ${h^{\prime }}_{1}>{h^{\prime }}_{2}$, therefore the Routh-Hurwitz conditions are satisfied and the equilibrium is stable [74]. If system (2) has two positive equilibriums, we denote them as (*p*_1_*,*h*_1_*) and (*p*_2_*,*h*_2_*) (without loss of generality, we suppose that *p*_1_* < *p*_2_* (see Fig 4). By the properties of *h*_1_(*p*) and *h*_2_(*p*), we can obtain ${h^{\prime }}_{1}\left(p_{2}{}^{*}\right)>{h^{\prime }}_{2}\left(p_{2}{}^{*}\right)$, (*p*_2_*)^2^ > *aδ*, $\text{det}(J(p_{2}^{*},h_{2}^{*}))>0,\text{tr}(J(p_{2}^{*},h_{2}^{*}))<0$, thus the equilibrium (*p*_2_*,*h*_2_*) is asymptotically stable. Whereas $\text{det}(J(p_{1}^{*},h_{1}^{*}))<0$ and the equilibrium (*p*_1_*,*h*_1_*) is a saddle point and unstable (see Fig 4).

## References

[1] Allee WC. Animal aggregations: a study in general sociology. University of Chicago Press, Chicago; 1931.

[2] Birkhead TR. The effect of habitat and density on breeding success in the common guillemot (Uria aalge). Journal of Animal Ecology. 1977; 46: 751–764.

[3] Dennis B. Allee effect: population growth, critical density, and the chance of extinction. Natural Resource Modeling. 1989; 3: 481–538.

[4] Lande R, Engen S, Sather BE. Stochastic Population Dynamics in Ecology and Conservation. Oxford University Press, Oxford; 2003.

[5] Sun GQ. Mathematical modeling of population dynamics with Allee effect. Nonlinear Dynamics. 2016; 85: 1–12.

[6] Sun GQ, Jin Z, Li L, Liu QX. The role of noise in a predator—prey model with Allee effect. Journal of Biological Physics. 2009; 35: 185–196. 10.1007/s10867-009-9139-y 19669561 PMC2669122

[7] Stephens PA, Sutherland WJ. Consequences of the Allee effect for behaviour, ecology and conservation. Tree. 1999; 14: 401–405. 10481204 10.1016/s0169-5347(99)01684-5

[8] Zhang F, Tao Y, Hui C. Organism-induced habitat restoration leads to bi-stability in metapopulations. Mathematical Biosciences. 2012; 240: 260–266. 10.1016/j.mbs.2012.08.006 22982509

[9] Liermann M, Hilborn R. Depensation: evidence, models and implications. Fish and Fisheries. 2001; 2: 33–58.

[10] Courchamp F, Berec L, Gascoigne J. Allee Effects in Ecology and Conservation. Oxford University Press, Oxford; 2008.

[11] Zu J, Mimura M. The impact of Allee effect on a predator-prey system with Holling type II functional response. Applied Mathematics and Computation. 2010; 217:3542–3556.

[12] Zhang F, Hui C, Han XZ, Li ZZ. Evolution of cooperation in patchy habitat under patch decay and isolation. Ecological Research. 2005; 20: 461–469.

[13] Zhang F, Hui C. Eco-evolutionary feedback and the invasion of cooperation in prisoner’s dilemma games. PLoS One. 2011; 6: e27523 10.1371/journal.pone.0027523 22125615 PMC3220694

[14] Berec L, Boukal DS, Berec M. Linking the Allee effect, sexual reproduction, and temperature-dependent sex determination via spatial dynamics. American Naturalist. 2001; 157: 217–230. 10.1086/318626 18707273

[15] Hui C, Li ZZ. Distribution patterns of metapopulation determined by Allee effects. Population Ecology. 2004; 46: 55–63.

[16] Charlesworth D, Charlesworth B. Inbreeding depression and its evolutionary consequences. Annual Review of Ecology and Systematics. 1987; 18: 237–268.

[17] Frankham R, Ballou JD, Briscoe DA. Introduction to Conservation Genetics. Cambridge University Press, Cambridge; 2002.

[18] Courchamp F, Clutton-Brock T, Grenfell B. Inverse density dependence and the Allee effect. Trends in Ecology and Evolution. 1999; 14: 405–410. 10481205 10.1016/s0169-5347(99)01683-3

[19] Stephens PA, Freckleton RP, Sutherland WJ. What is the Allee effect? Oikos. 1999; 87: 185–190.

[20] Hammond DS. Post-dispersal seed and seeding dispersal in tropical dry forest trees after shifting agriculture, Chiapas, Mexico. Journal of Tropical Ecology. 1995; 11: 295–313.

[21] Amarasekare P. Allee effects in metapopulation dynamics. American Naturalist. 1998; 152: 298–302. 10.1086/286169 18811393

[22] Hanski I. Metapopulation Ecology. Oxford University Press, Oxford; 1999.

[23] Zhou SR, Wang G. Allee-like effects in metapopulations dynamics. Mathematical Biosciences. 2004; 189:103–113. 10.1016/j.mbs.2003.06.001 15051417

[24] Berec L, Angulo E, Courchamp F. Multiple Allee effects and population management. Trends in Ecology and Evolution. 2007; 22: 185–191. 10.1016/j.tree.2006.12.002 17175060

[25] Ferson S, Burgman MA. The dangers of being few: demographic risk analysis for rare species extinction. New York State Museum Bulletin. 1990; 471: 129–132.

[26] Begon M, Harper JL, Townsend CR. Ecology: Individuals, Populations and Communites. Blackwell, Oxford; 1996

[27] Jones CG, Lawton JH, Schachak M. Organisms as ecosystem engineers. Oikos. 1994; 69: 373–386.

[28] Hastings A, Byers JE, Crooks JA, Cuddington K, Jones CG, Lambrinos JG, et al Ecosystem engineering in space and time. Ecology Letters, 2007; 10: 153–164. 10.1111/j.1461-0248.2006.00997.x 17257103

[29] Post DM, Palkovacs EP. Eco-evolutionary feedbacks in community and ecosystem ecology: interactions between the ecological theatre and the evolutionary play. Philosophical Transactions of the Royal Society. Series B: Biological Sciences. 2009; 364: 1629–1640. 10.1098/rstb.2009.0012 PMC2690506 19414476

[30] Seto M, Iwasa Y. Regime shift and robustness of organism-created environments: A model for microbial ecosystems. Journal of Theoretical Biology. 2011; 269: 297–306. 10.1016/j.jtbi.2010.10.035 21055409

[31] Wertheim B, Marchais J, Vet LEM, Dicke M. Allee effect in larval resource exploitation in Drosophila: an interaction among density of adults, larvae, and micro-organisms. Ecological Entomology. 2002; 27: 608–617.

[32] Gascoigne J, Beadman HA, Saurel C, Kaiser MJ. Density dependence, spatial scale and patterning in sessile biota. Oecologia. 2005; 145: 371–381. 10.1007/s00442-005-0137-x 15968539

[33] Levins R. Some Demographic and Genetic Consequences of Environmental Heterogeneity for Biological Control. Bulletin of the ESA. 1969; 15: 237–240.

[34] McVinish R, Pollett PK. Interaction between habitat quality and an Allee-like effect in metapopulation. Ecological Modelling. 2013; 249: 84–89.

[35] Zhou SR, Liu CZ, Wang G. The competitive dynamics of metapopulations subject to the Allee-like effect. Theoretical Population Biology. 2004; 65: 29–37. 14642342 10.1016/j.tpb.2003.08.002

[36] Zhou SR, Liu YF, Wang G. The stability of predator-prey systems subject to the Allee effects. Theoretical Population Biology. 2005; 67: 23–31. 10.1016/j.tpb.2004.06.007 15649521

[37] Wang G, Liang XG, Wang FZ. The competitive dynamics of populations subject to an Allee effect. Ecological Modelling. 1999; 124: 183–192.

[38] Petrovskii SV, Li BL. Exactly solvable models of biological invasion. Chapman & Hall/CRC, Boca Raton; 2006.

[39] Zhang F, Li ZZ, Hui C. Spatiotemporal dynamics and distribution patterns of cyclic competition in metapopulation. Ecological Modelling. 2006; 193: 721–735.

[40] Dieckmann U, Law R, Metz JAJ. The Geometry of Ecological Interactions: Simplifying Spatial Complexity. Cambridge University Press, Cambridge; 2000.

[41] Hui C, Li ZZ. Dynamical complexity and metapopulation persistence. Ecological Modelling. 2003; 164: 201–210.

[42] Byers JE, Cuddington K, Jones CG, Talley TS, Hastings A, Lambrinos JG, et al Using ecosystem engineers to restore ecological systems. Trends in Ecology and Evolution. 2006; 21:493–500. 10.1016/j.tree.2006.06.002 16806576

[43] Yue DX, Hui C, Li ZZ. Niche construction for desert plants in individual and population scales: theoretical analysis and evidences from saksaul (Haloxylon ammodendron) forests. Israel journal of plant sciences. 2004; 52: 235–244.

[44] Zarnetske PL, Hacker SD, Seabloom EW, Ruggiero P, Killian JR, Maddux TB, et al Biophysical feedback mediates effects of invasive grasses on coastal dune shape. Ecology. 2012; 93:1439–1450. 22834384 10.1890/11-1112.1

[45] Hui C, Li ZZ, Yue DX. Metapopulation dynamics and distribution, and environmental heterogeneity induced by niche construction. Ecological Modelling. 2004; 177: 107–118.

[46] Murray JD. Mathematical Bilolgy. Springer, Berlin; 2003.

[47] Rietkerk M, Boerlijst M, van Langevelde F., Hillerislambers R, de Koppel J, Kumar L, et al Self-organisation of vegetation in arid ecosystems. American Naturalist. 2002; 160: 524–530. 10.1086/342078 18707527

[48] Rietkerk M, Dekker S, de Ruiter P, van de Koppel J. Self-organized patchiness and catastrophic shifts in ecosystems. Science. 2004; 305: 1926–1929. 10.1126/science.1101867 15448261

[49] Gascoigne J, Lipcius RN. Periodic dynamics in a two-stage Allee effect model are driven by tension between stage equilibria. Theoretical Population Biology. 2005; 68: 237–241. 10.1016/j.tpb.2005.02.005 15949831

[50] Berryman AA. On principles, laws and theory in population ecology. Oikos. 2003; 103:695–701.

[51] Eskola HTM, Parvinen K. On the mechanistic underpinning of discrete-time population models with Allee effect. Theoretical Population Biology. 2007; 72: 41–51. 10.1016/j.tpb.2007.03.004 17467760

[52] Molnár PK, Lewis MA, Derocher AE. Estimating Allee dynamics before they can be observed: polar bears as a case study. PLoS ONE. 2014; 9: 1–12. 10.1371/journal.pone.0085410 PMC3888426 24427306

[53] Tayor CM, Hastings A. Allee effects in biological invasions. Ecology Letters. 2005; 8: 895–908.

[54] Stephens PA. Two may be company but three is seldom a crowd: Allee Effects in Ecology and Conservation. Conservation Biology. 2008; 22: 1662–1664.

[55] Kramer AM, Dennis B, Liebhod AM, Drake JM. The evidence for Allee effects. Population Ecology. 2009; 51: 341–354.

[56] Sun GQ, Wu ZY, Wang Z, Jin Z. Influence of isolation degree of spatial patterns on persistence of populations. Nonlinear Dynamics. 2016; 83: 811–819.

[57] Sun GQ, Chakraborty A, Liu QX, Jin Z, Anderson KE, Li BL. Influence of time delay and nonlinear diffusion on herbivore outbreak. Commun Nonlinear Sci Numer Simulat. 2014; 19: 1507–1518.

[58] Li L, Jin Z. Pattern dynamics of a spatial predator—prey model with noise. Nonlinear Dynamics. 2012; 67: 1737–1744.

[59] Sun GQ, Jusup M, Jin Z, Wang Y, Wang. Pattern transitions in spatial epidemics: Mechanisms and emergent properties. Physics of Life Reviews. 2016; 19: 43–73. 10.1016/j.plrev.2016.08.002 27567502 PMC7105263

[60] Sun GQ, Zhang J, Song LP, Jin Z, Li BL. Pattern formation of a spatial predator—prey system. Applied Mathematics and Computation. 2012; 218: 11151–11162.

[61] Li L, Jin Z, Li J. Periodic solutions in a herbivore-plant system with time delay and spatial diffusion. Applied Mathematical Modelling. 2016; 40: 4765–4777.

[62] Sun GQ, Wang SL, Ren Q, Jin Z, Wu YP. Effects of time delay and space on herbivore dynamics: linking inducible defenses of plants to herbivore outbreak. Scientific Reports. 2015; 5: 11246 10.1038/srep11246 26084812 PMC4471659

[63] Tilman D. Competiton and biodiversity in spatially structured habitats. Ecology. 1994;75: 2–16.

[64] Snyder RE. Spatiotemporal population distributions and their implications for species coexistence in a variable environment. Theoretical Population Biology. 2007; 72: 7–20. 10.1016/j.tpb.2007.03.009 17499323

[65] Murrell DJ. When does local spatial structure hinder competitive coexistence and reverse competitive hierarchies? Ecology. 2010; 91: 1605–1616. 20583703 10.1890/09-0832.1

[66] Killingback T, Blok HJ, Doebeli M. Scale-free extinction dynamics in spatially structured host-parasitoid systems. Journal of Theoretical Biology. 2006; 241: 745–750. 10.1016/j.jtbi.2006.01.010 16487977

[67] Brandt AJ, Seabloom EW. Seed and establishment limitation contribute to long-term native forb declines in California grasslands. Ecology. 2012; 93: 1451–1462. 22834385 10.1890/11-0579.1

[68] Rass L. Pandemic bounds for an epidemic on an infinite lattice. Mathematical Biosciences. 2005; 195: 194–209. 10.1016/j.mbs.2005.02.005 15921705

[69] Tuckwell HC, Toubiana L. Dynamical modeling of viral spread in spatially distributed populations: stochastic origins of oscillations and density dependence. Biosystems. 2007; 90: 546–559. 10.1016/j.biosystems.2006.12.006 17324498 PMC7115796

[70] Hanert E, Schumacher E, Deleersnijder E. Front dynamics in fractional-order epidemic models. Journal of Theoretical Biology. 2011; 279: 9–16. 10.1016/j.jtbi.2011.03.012 21420979

[71] Shaw MW. The population dynamics of disease on short and long time-scales. European Journal of Plant Pathology. 2014; 138: 487–497.

[72] Nowak MA, May RM. Evolutionary games and spatial chaos. Nature. 1992; 359: 826–829.

[73] Nicholas JS, Paulien H. Spatially induced speciation prevents extinction: the evolution of dispersal distance in oscillatory predator-prey models. Proceedings of the Royal Society B: Biological Sciences. 1998; 265: 25–32. 10.1098/rspb.1998.0259 9470215 PMC1688752

[74] Kot M. Elements of Mathematical Ecology. Cambridge University Press, Cambridge; 2001.

